# Extensive ischemic ulcers due to limb occlusion after endovascular aneurysm repair: a case report

**DOI:** 10.1186/s40064-016-2345-8

**Published:** 2016-06-18

**Authors:** Yoshito Kadoya, Tsuneaki Kenzaka, Daisuke Naito

**Affiliations:** Department of Internal Medicine, Kyotambacho Hospital, 28 Wadaooshita, Kyotambacho, Funai, Kyoto 622-0311 Japan; Division of Community Medicine and Career Development, Kobe University Graduate School of Medicine, Kobe, Japan; Department of Cardiology, Fukuchiyama City Hospital, Fukuchiyama, Kyoto Japan

**Keywords:** Case reports, Complications, Endovascular procedures

## Abstract

**Introduction:**

Limb occlusion after endovascular aneurysm repair (EVAR) is a well-known complication. However, extensive ischemic ulcers due to limb occlusion are extremely rare.

**Case description:**

We report a rare case of extensive ischemic ulcers that developed seven months after EVAR in an 85-year-old Japanese man. He had been taking appropriate anticoagulant therapy because of paroxysmal atrial fibrillation. Angiography showed a left limb occlusion and superficial femoral artery (SFA) chronic total occlusion (CTO), and intravascular ultrasound showed limb kinking. Endovascular therapy (EVT) was performed, and stent placement was used to cover a large amount of thrombi and correct the limb kinking, leading to complete recovery of left limb blood flow. After additional EVT was performed for the SFA CTO, outflow improved and the ulcers healed completely.

**Discussion and evaluation:**

It seemed that the combination of poor inflow and poor outflow led to limb thrombosis.

**Conclusions:**

Here, we describe an extremely rare case of extensive ischemic ulcers due to limb occlusion after EVAR. Patients should undergo careful follow-up after EVAR to monitor blood flow to the lower extremities. Additionally, the early detection and correction of limb kinking and poor outflow are essential to prevent the development of ischemic ulcers.

## Background

Endovascular aneurysm repair (EVAR) has been widely used for treating patients with abdominal aortic aneurysm (AAA). EVAR is similar to open repair in terms of long-term mortality, but it has a higher rate of re-intervention (United Kingdom EVAR Trial Investigators 2010). Limb occlusion after EVAR is a well-known complication that requires re-intervention (EVAR trial participants 2005; Sampram et al. 2003). However, ischemic ulcers due to limb occlusion are very rare. Here, we report an extremely rare case of extensive ischemic ulcers due to limb occlusion after EVAR.

### Case presentation

**Fig. 1:**
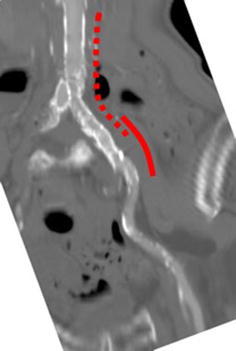
Computed tomography findings. There was no limb stenosis or kinking after endovascular aneurysm repair (*dotted line* left graft limb, *solid line*, SMART^®^ stent)

An 85-year-old Japanese man underwent EVAR for an AAA at another hospital in June 2014. Graft limbs were placed in both the right and left common iliac arteries (CIAs). During post-dilatation, the Equalizer^®^ balloon catheter (Boston Scientific) ruptured and was trapped in the left CIA due to severe calcification, and a part of this catheter remained in the left CIA. A SMART^®^ stent (10 × 40 mm; Cordis Endovascular) was also implanted in the left CIA to cover the remaining balloon. Thereafter, post-dilatation was performed again with a percutaneous transluminal angioplasty balloon (10 mm), and final angiography showed good left limb blood flow. Computed tomography after EVAR showed no limb stenosis or kinking (Fig. 1).

**Fig. 2:**
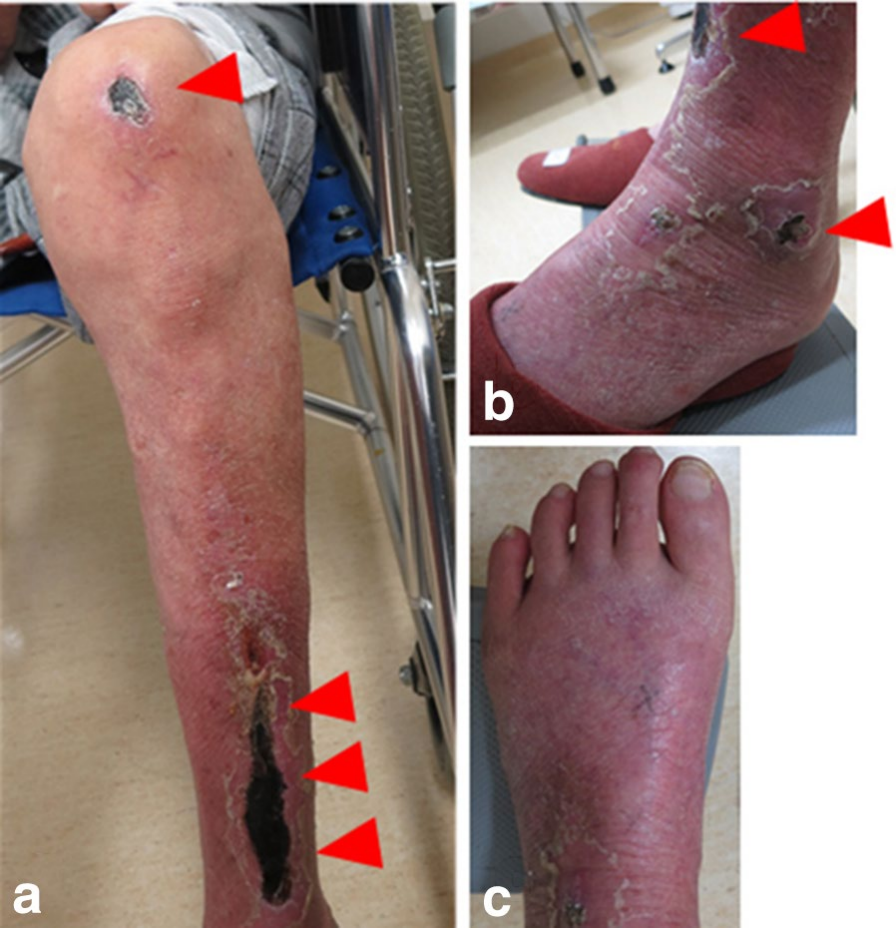
Photograph of the patient. Ulcers were present on the left leg (*arrowheads*) 7 months after endovascular aneurysm repair (**a** knee and lower leg, **b** ankle, **c** foot)

**Fig. 3:**
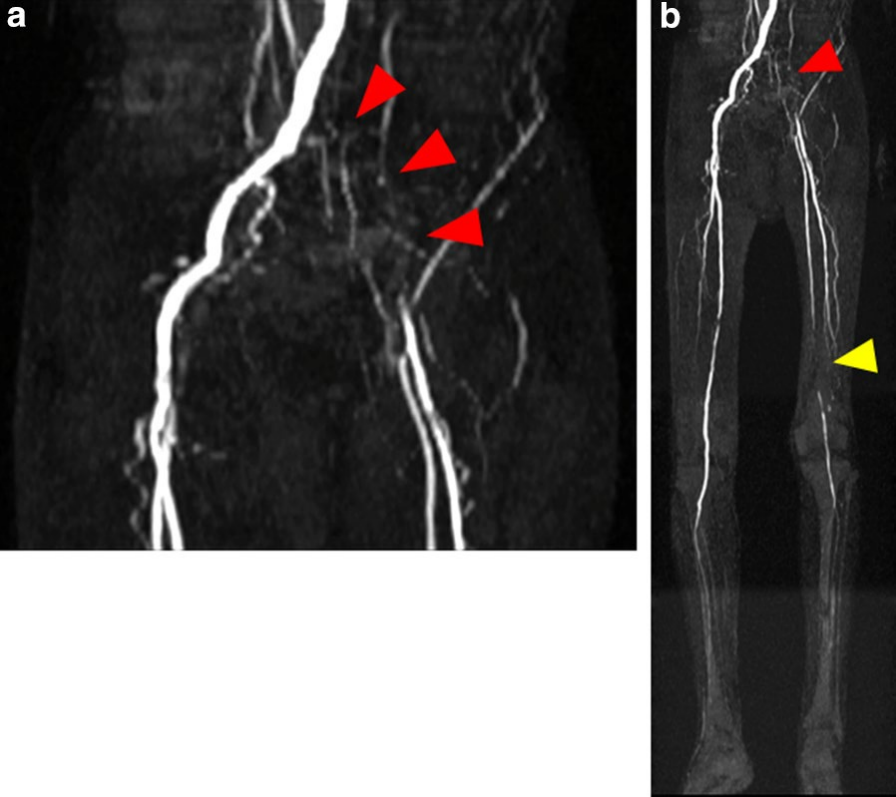
Magnetic resonance angiography findings. A left graft limb occlusion (*red arrowheads*) and left superficial femoral artery chronic total occlusion (*yellow arrowhead*) were observed (**a** pelvic region, **b** all lower limb regions)

The patient developed left intermittent claudication in late January 2015. His symptom developed gradually, not suddenly. Subsequently, he experienced rest pain and developed ulcers on his left leg; he visited our hospital at the beginning of February 2015 (Fig. 2a–c). He had a history of smoking with a Brinkman index score of 200. His medical history included an old myocardial infarction, congestive heart failure, cerebral infarction, and paroxysmal atrial fibrillation. The patient was taking appropriate anticoagulant therapy with warfarin (3 mg/day) and antiplatelet therapy with aspirin (100 mg/day). The prothrombin time and international normalized ratio were well controlled. The laboratory findings were as follows: a white blood cell count of 6670/μL; hemoglobin, 12.2 g/dL; platelets, 193,000/μL; total bilirubin, 1.0 mg/dL; aspartate aminotransferase, 20 IU/L; alanine aminotransferase, 7 IU/L; lactic dehydrogenase, 263 IU/L; blood urea nitrogen, 27 mg/dL; creatinine, 1.75 mg/dL; prothrombin time/international normalized ratio, 2.65; activated partial thromboplastin time, 54.1 s. The ankle-brachial index (ABI) of the left leg could not be measured. The skin perfusion pressure was approximately 40 mmHg in both the dorsalis pedis and plantar arteries. Magnetic resonance angiography and ultrasonography showed a left graft limb occlusion and left superficial femoral artery (SFA) occlusion. The deep femoral artery was patent, and it supplied collateral blood flow to the popliteal artery; however, arteries below the knee were totally occluded (Fig. 3a, b). The lower limb blood flow had not been evaluated before EVAR; however, the rich collateral vessels from the deep femoral to the popliteal artery indicated that the SFA occlusion was a chronic lesion. Therefore, the patient was diagnosed with ischemic ulcers, i.e., critical limb ischemia (Rutherford 5), due to left limb occlusion and SFA chronic total occlusion (CTO). Embolization due to atrial fibrillation was less likely to be the cause in this case, because the ischemic symptoms had developed chronically, not acutely; in addition, the patient had been receiving appropriate anticoagulant therapy, and he had no recent episode of atrial fibrillation. We discussed a treatment strategy, including thrombolysis, surgical thrombectomy, and femoral–femoral or axillo-femoral bypass grafts. For thrombolysis, anticoagulant therapy was concomitantly used, and the patient was considered to have a higher risk of hemorrhagic complications. Furthermore, as the patient was unwilling to undergo surgical treatment, open surgery was not considered. Moreover, taking into account the patient’s condition (i.e., low left ventricular function and critical limb ischemia), we initially administered EVT to treat the left limb occlusion without thrombolysis.

**Fig. 4:**
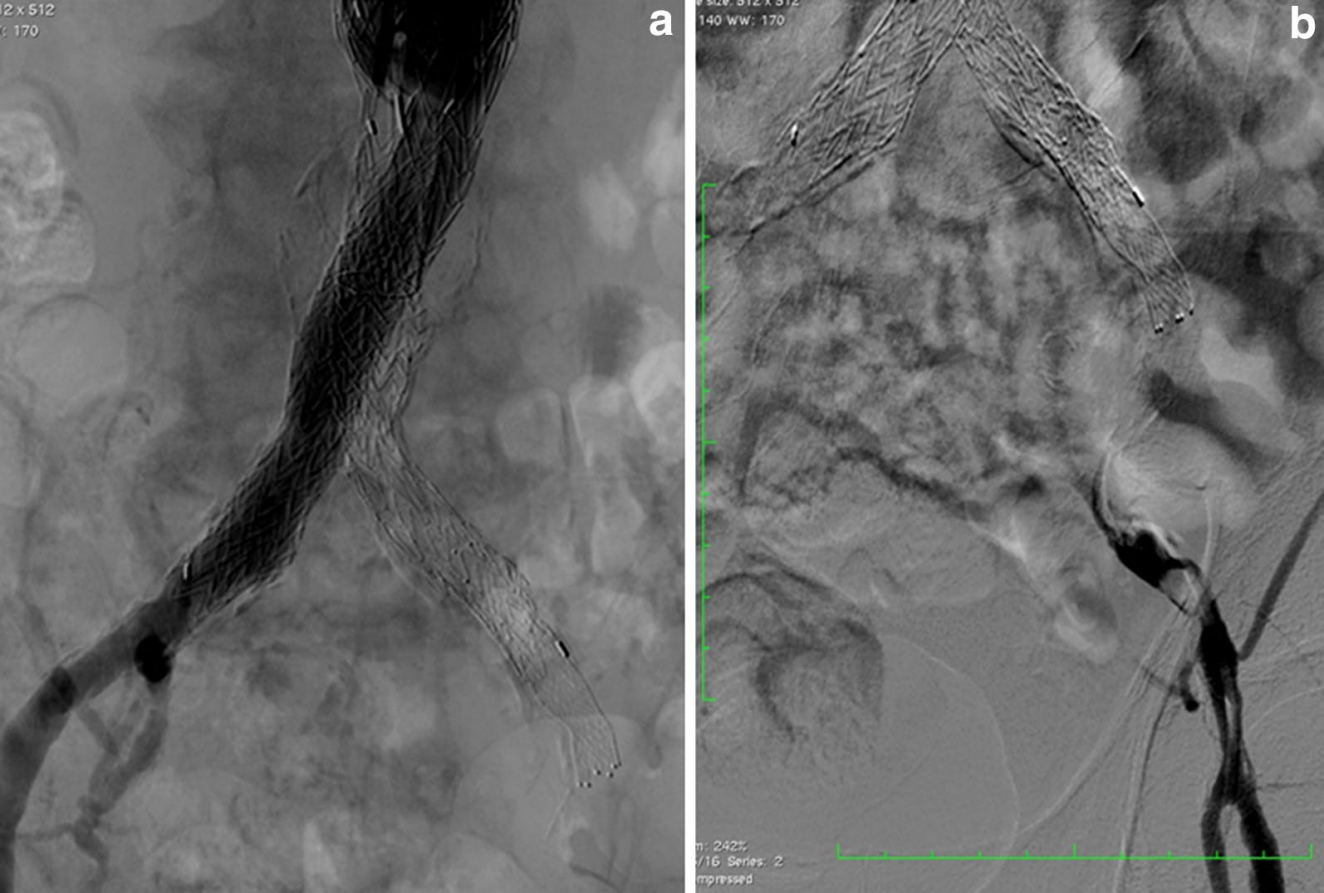
Initial angiography findings. The left graft limb was totally occluded with a large amount of thrombi (**a** antegrade angiography, **b** retrograde angiography)

**Fig. 5:**
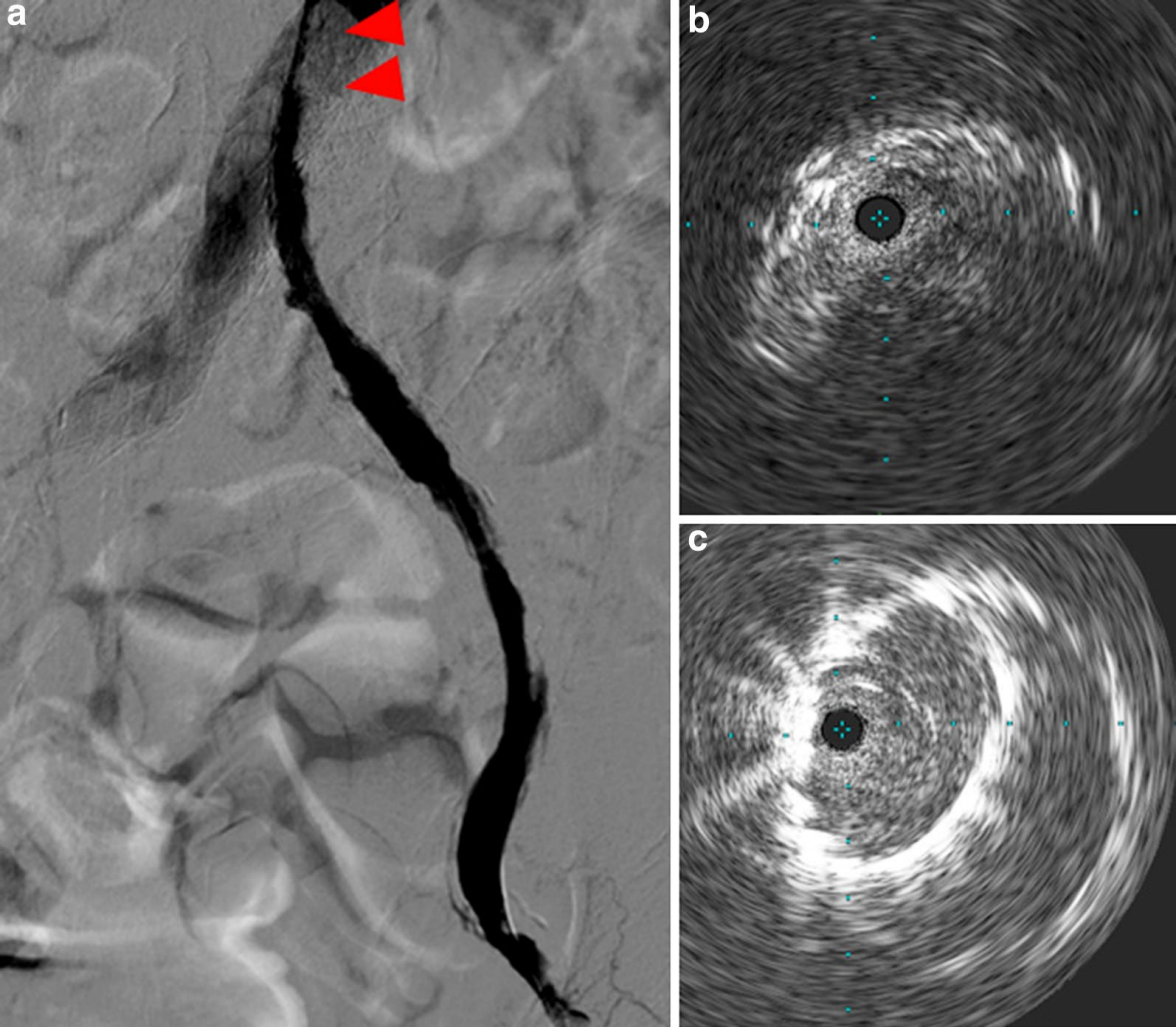
**a** Angiography findings. The entry of the left limb still seemed narrow after pre-dilatation (*arrowhead*). **b** Intravascular ultrasonography findings. The lumen was deformed into a crescent-moon shape. **c** Intravascular ultrasonography findings. A decrease in the deformation was observed after stent implantation

**Fig. 6:**
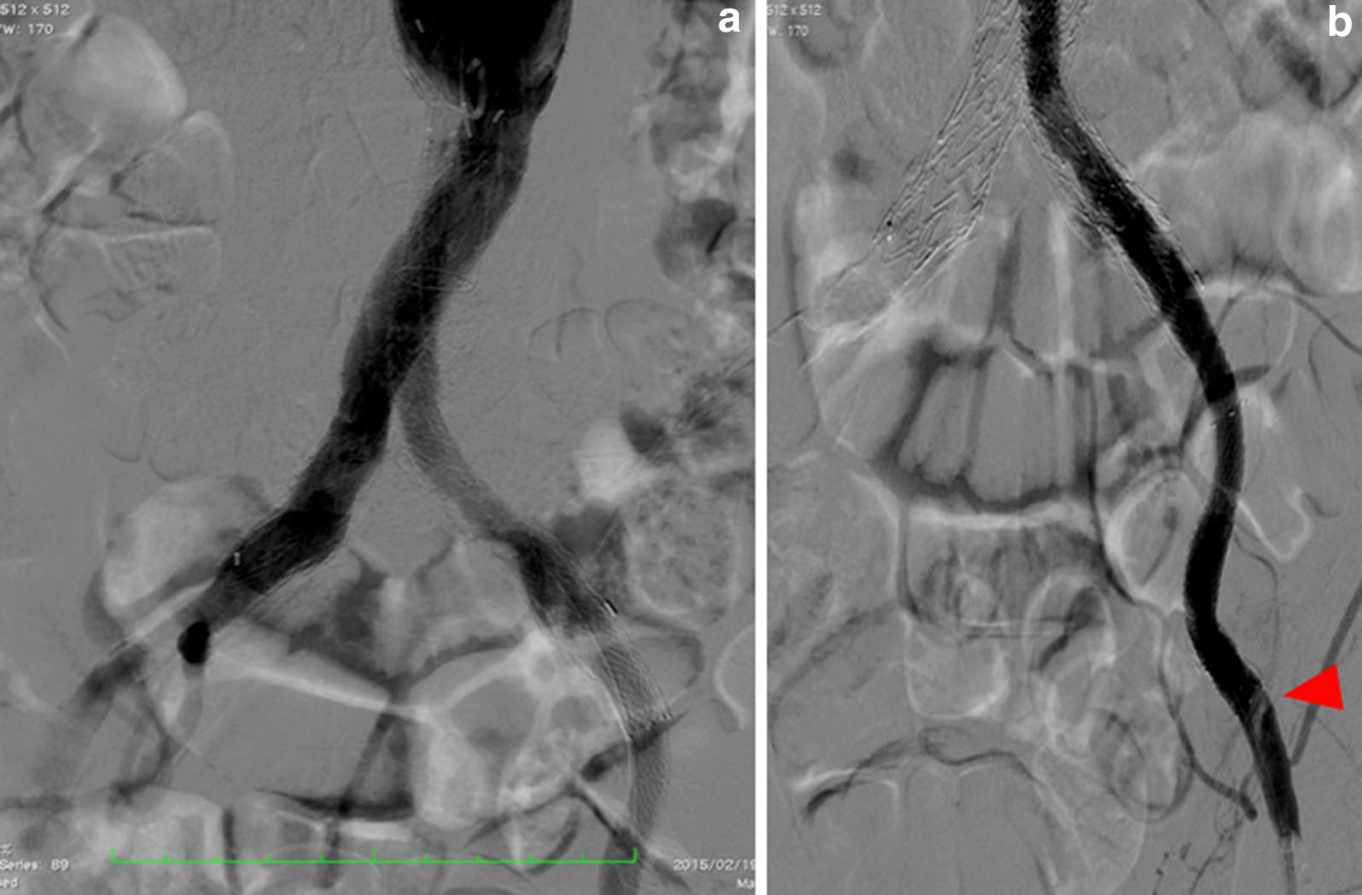
Angiography findings. Although a small amount of thrombi was found in the left common femoral artery (*arrowhead*), the final angiography results showed good antegrade flow (**a** antegrade angiography, **b** retrograde angiography)

**Fig. 7:**
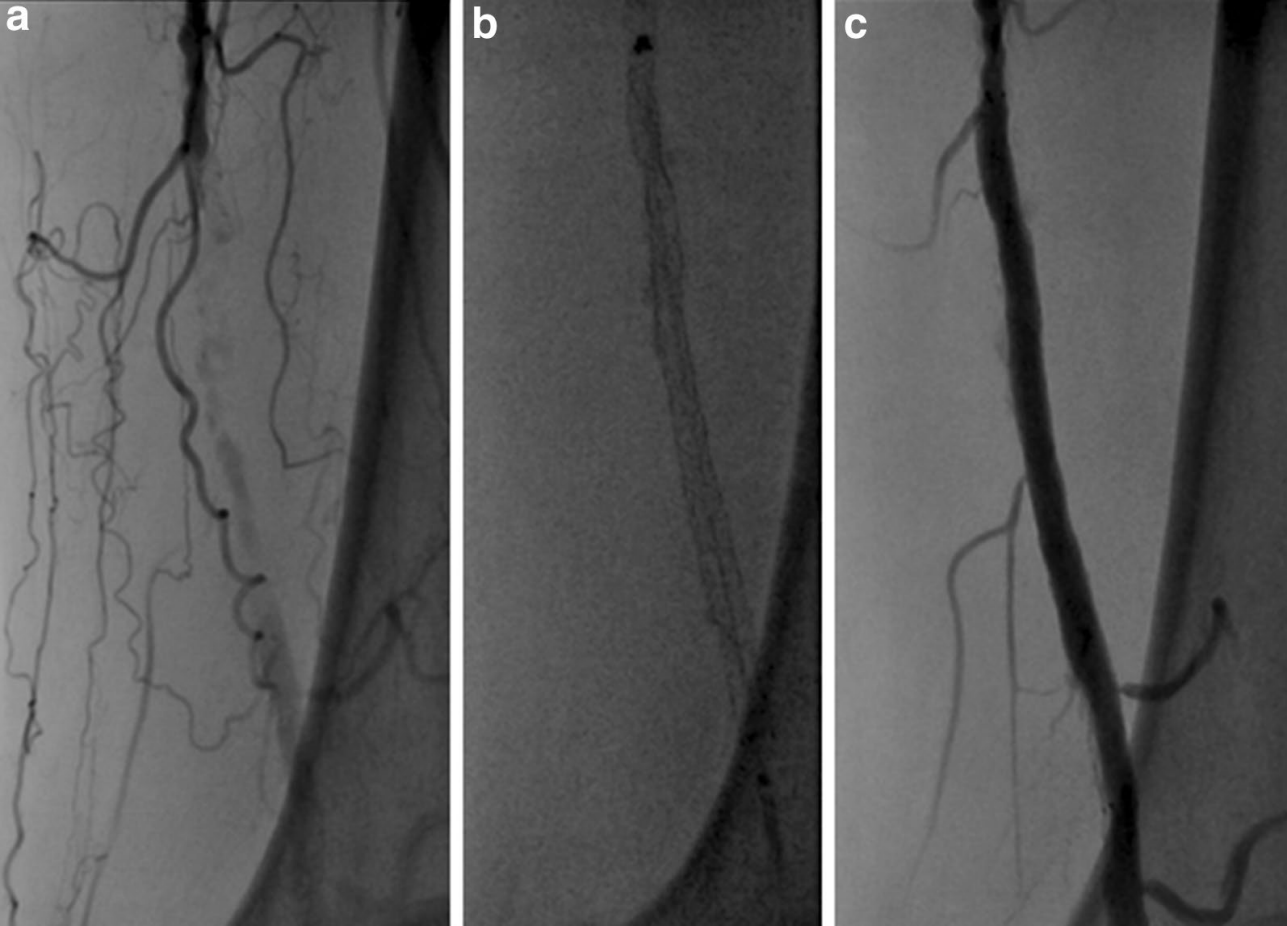
Angiographic images of endovascular therapy (EVT). EVT for the left superficial femoral artery chronic total occlusion was performed 2 weeks after EVT for the left limb occlusion (**a** initial angiography, **b** stenting, **c** final angiography)

Initial angiography showed that the left graft limb was totally occluded with a large amount of thrombi (Fig. 4a, b) and there was severe calcification in the external iliac artery (EIA). A 9-French (Fr) Optimo^®^ occlusion catheter (Tokai Medical Products) was inserted via the left femoral artery to prevent peripheral embolization, and we attempted to pass wires through the lesion. We changed the wires several times because the EIA had stenosis and calcification, and finally, the wires were passed through the lesion into the aorta. Thereafter, thrombus aspiration was performed by using a 6-Fr guiding catheter, and a large amount of red thrombus was aspirated. Pre-dilatation was performed with 6- and 8-mm balloons. A major portion of the thrombi present were removed; however, antegrade blood flow was poor. Furthermore, the entry of the left limb still seemed narrowed (Fig. 5a). Unfortunately, retrograde angiography performed a few minutes later showed thrombotic re-occlusion of the left limb. Thrombus aspiration and balloon angioplasty were performed repeatedly, but a large amount of thrombi was uncontrolled. Therefore, we placed a SMART^®^ stent (8 × 100 mm; Cordis Endovascular) in the left EIA to cover the remaining thrombi, and the thrombi were consequently controlled. We examined the narrow entry of the left limb by using intravascular ultrasound (IVUS), and we found that the lumen was deformed into a crescent-moon shape (Fig. 5b). The balloon expansion was also poor. Based on the IVUS image and balloon expansion, we found a kink in the limb at the entry of the left limb. Therefore, we placed an additional SMART^®^ stent (10 × 60 mm; Cordis Endovascular) to correct the limb kinking. After stenting, IVUS showed reduction of the deformation (Fig. 5c). A small amount of thrombi remained in the left common femoral artery; however, final angiography showed good antegrade flow, and we completed the procedure (Fig. 6a, b). The patient continued anticoagulant therapy with warfarin (3 mg/day) and antiplatelet therapy with aspirin (100 mg) for life. After the first EVT, the ABI improved to 0.65. Two weeks later, the second EVT for the left SFA CTO was performed (Fig. 7a–c). The ABI finally improved from 0.65 to 0.83. Ulcers on the left leg had completely healed after debridement and skin grafting.

**Fig. 8:**
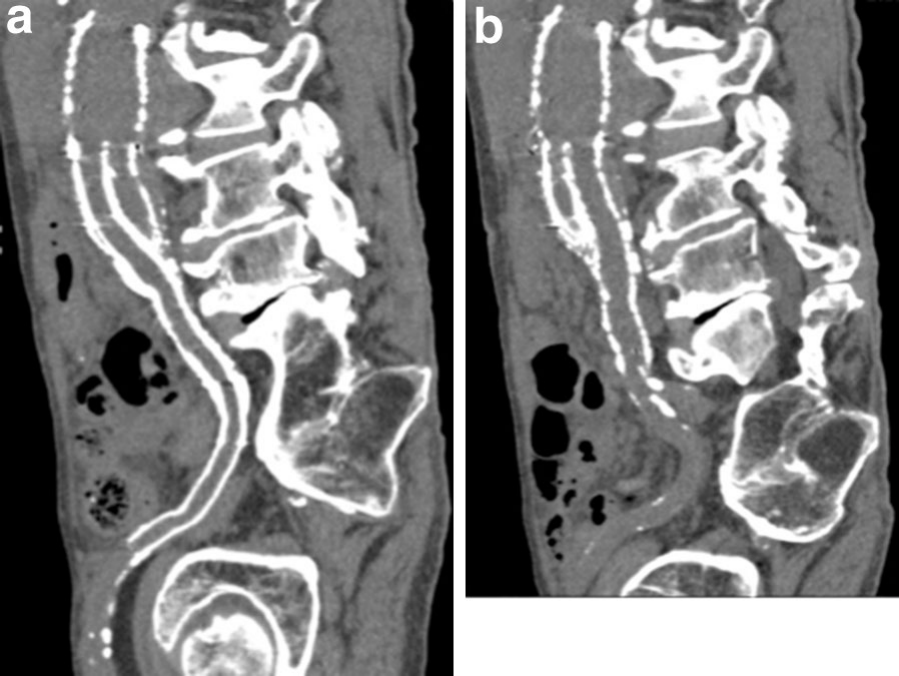
Computed tomography angiography findings. Good expansion of the two implanted stents was observed. No kink or stenosis was observed in either limb (**a** the left limb, **b** the right limb)

Seven months after the first EVT, the ABI of the left leg improved to 0.96, and computed tomography angiography showed good expansion of the two implanted stents and no kinks or stenosis in either limb (Fig. 8a–b). We continue to follow-up this patient regularly at our hospital, and he has had no signs of recurrence for 12 months.

## Conclusions

We experienced an extremely rare case of extensive ischemic ulcers due to limb occlusion after EVAR. Limb occlusion is a well-known complication occurring in 2.6–7.4 % of patients during follow-up after EVAR (Sampram et al. 2003, Fairman et al. 2002, Ronsivalle et al. 2013), whereas the development of ischemic ulcers is very rare. Cochennec et al. reported that 27 % of patients present with acute ischemia, 24 % with rest pain, 30 % with claudication, and 12 % with no symptoms (Cochennec et al. 2007). Mantas et al. reported that 39 % of patients present with acute ischemia, and the remaining 61 % present with claudication (Mantas et al. 2015). We could not find any other cases of ischemic ulcers due to limb occlusion after EVAR. To the best of our knowledge, this is the first reported case of this rare condition.

Several predictive factors of limb occlusion have been proposed. Anatomic factors include angulation, calcification, and tortuosity of the iliac arteries. Graft-related factors include limb kinking, limb oversizing, and extension to the EIA (Cochennec et al. 2007; Mantas et al. 2015; Woody and Makaroun 2004). Poor outflow also increases the risk of limb thrombosis (Cochennec et al. 2007). The present case had limb kinking and SFA CTO; therefore, we thought that the combination of poor inflow and poor outflow led to the limb thrombosis. The possibility of distal embolization due to atrial fibrillation could not be completely denied, but this seemed less likely because the patient developed left leg ischemia chronically, not acutely, and the patient had been receiving appropriate anticoagulant therapy, ensuring good control.

To prevent the development of ischemic ulcers due to limb occlusion, patients who have undergone EVAR should be carefully followed-up to monitor blood flow to the lower extremities. In our case, limb kinking and SFA CTO was identified as a significant cause of limb occlusion. As for the limb kinking, it occurred sometime after the EVAR, not during EVAR or immediately after EVAR, since computed tomography immediately after EVAR showed no limb stenosis or kinking. Troisi et al. reported limb stenosis or kinking in 12.2 % of patients during EVAR, with implantation of a metallic stent reducing the occurrence of limb occlusion to 0.9 % (Troisi et al. 2010). Otherwise, Carpenter et al. suggested that limb kinking occurs due to aneurysm sac shrinkage after EVAR (Carpenter et al. 2002). Therefore, making an accurate prediction of limb kinking is quite challenging. Moreover, as for the SFA CTO, because the lower limb blood flow had not been evaluated before EVAR, it was not recognized. Another possibility was that the severe SFA stenosis developed into total occlusion after EVAR. Nevertheless, the SFA lesion should be recognized and treated before EVAR. If we had been able to detect limb kinking and SFA CTO before limb occlusion, the development of ischemic ulcers would have been prevented. Thus, careful follow-up for patients after EVAR is important, and early detection and correction of limb kinking and poor outflow are essential to prevent the development of ischemic ulcers due to limb occlusion.

Regarding treatment, both surgical and endovascular options are available. However, the optimal treatment of limb occlusion is unclear (Cochennec et al. 2007; Mantas et al. 2015; Woody and Makaroun 2004). Surgical options, including thrombectomy and extra-anatomic bypass graft, are associated with the possibility of graft damage or dislodgment and endoleaks during thrombectomy (Woody and Makaroun 2004). For patients who have critical limb ischemia with a serious comorbidity such as low left ventricular function, EVT may be a less invasive option for limb occlusion.

In conclusion, we describe an extremely rare case of extensive ischemic ulcers due to limb occlusion after EVAR. After EVAR, patients should undergo careful follow-up to monitor blood flow to the lower extremities. Additionally, the early detection and correction of limb kinking and poor outflow are essential to prevent the development of ischemic ulcers.

## Abbreviations

EVAR: endovascular aneurysm repair
AAA: abdominal aortic aneurysm
CIA: common iliac artery
ABI: ankle-brachial index
SFA: superficial femoral artery
CTO: chronic total occlusion
IVUS: intravascular ultrasound
EVT: endovascular therapy
EIA: external iliac artery
Fr: French
